# The efficacy of music therapy in alleviating anxiety among college students: a systematic review and meta-analysis

**DOI:** 10.3389/fpsyg.2025.1723316

**Published:** 2026-02-09

**Authors:** Sirong Li, Zixuan Gong, Rujie Wang, Jialing Liu, Xingxin Zhan, Jia Lan, Hui Feng

**Affiliations:** 1 Department of Health Promotion and Health Intervention, School of Public Health, Xinyu University, Xinyu, Jiangxi, China; 2 School of Music, Xinyu University, Xinyu, Jiangxi, China

**Keywords:** anxiety, college students, meta-analysis, music therapy, systematic review

## Abstract

**Background:**

Anxiety is a prevalent psychological issue among university students, adversely affecting their mental health and academic performance. Music therapy (MT) has emerged as a promising non-pharmacological intervention. This systematic review and meta-analysis aims to evaluate MT’s efficacy in alleviating anxiety symptoms among college students.

**Methods:**

A comprehensive search of eight databases was conducted up to December 1, 2025.Randomized controlled trials (RCTs) involving MT interventions for anxiety reduction in college students were included. Study selection, data extraction, and quality assessment using the Cochrane Risk of Bias tool were performed independently by two reviewers. Meta-analysis was conducted using RevMan 5.2 and Stata 16.0.

**Results:**

Eighteen RCTs (from 17 included studies) involving 1,679 participants (829 in the experimental group; 850 in the control group) were included. The meta-analysis demonstrated that MT significantly alleviated anxiety symptoms compared to control groups [standardized mean difference (SMD) = –1.54, 95% confidence interval (CI): –2.08 to –1.01, *P* < 0.00001] with high heterogeneity (*I*^2^ = 95%). Subgroup analyses revealed that both passive and combined (active and passive) MT were effective, with combined interventions showing a larger effect. Studies conducted in domestic settings (*n* = 13) showed a more significant effect than international ones (*n* = 3). Significant anxiety reduction was observed across both short-term (<8 weeks) and long-term ( ≥ 8 weeks) interventions.

**Conclusion:**

MT is an effective intervention for alleviating anxiety in university students, with combined delivery formats and longer durations showing particular promise. It represents a safe, low-cost, and flexible option for campus mental health services. Future research should prioritize standardizing MT protocols, implementing long-term follow-ups, and conducting cross-cultural validations to facilitate its broader integration.

## 1 Introduction

The World Health Organization (WHO) defines mental health as “not merely the absence of disease or infirmity, but a state of complete physical, mental, and social wellbeing (International Health Conference, 2003).”However, contemporary college students face multiple challenges such as academic competition (Posselt and Lipson, 2016), social pressure (Leary and Kowalski, 1995), career uncertainty (Herr, 2001), and “involution” (Yi et al., 2022), making anxiety a common psychological issue among this group. These factors contribute to the high prevalence of anxiety disorders within this population (Tan et al., 2023). Surveys indicate that approximately 38–43% of college students in China experience symptoms of anxiety, while 20–30% report high levels of stress (Hongjie, 2019; Browning et al., 2023; Li et al., 2023; Liu et al., 2023). This issue represents a significant global public health concern. For instance, a recent meta-analysis indicates that the pooled prevalence of anxiety symptoms among college students is 39.0% (95% CI: 34.6–43.4%), with particularly high rates observed in North America (48.3%, 95% CI: 37.4–59.2%) and lower middle-income countries (54.2%, 95% CI: 35.0–73.4%) (Li et al., 2022). At a university in Saudi Arabia, a Middle Eastern nation, 29.5% of students experience moderate to severe depression, while 39% contend with moderate, severe, or very severe anxiety (Al-Garni et al., 2025). According to the findings of the World Health Organization World Mental Health International Collaborative Survey (WHO WMH-ICS),35 % of first-year college students met the diagnostic criteria for at least one mental disorder, with major depressive disorder (MDD) and generalized anxiety disorder (GAD) being the most prevalent (Auerbach et al., 2018). These data collectively demonstrate that anxiety and depression are not products of a single culture or educational system, but rather a widespread phenomenon across regions. Persistent anxiety not only reduces college students’ sense of happiness and causes physical and mental exhaustion (Yangqian, 2021), but also seriously impairs social functions, manifested as declining academic performance, suspension, dropout, and even self-harm or suicide (Jinchen et al., 2025), directly affecting the quality of talent cultivation. In the long run, untreated anxiety may persist into adulthood, increasing the risk of complications of generalized anxiety disorder (such as depression), thereby affecting treatment response and outcomes (Noyes, 2001).

Currently, intervention methods for college students’ anxiety mainly fall into two categories: pharmacological intervention and non-pharmacological intervention. Pharmacotherapy, on the other hand, may produce side effects of varying degrees (Bourin et al., 1993). Non-pharmacological intervention include an enriched environment, regular exercise, and music (Taheri Zadeh et al., 2021), muscle relaxation, Internet-based integrated intervention (Ding et al., 2021). Among these, music therapy is gradually showing unique application value and development potential. As a non-invasive intervention, music offers unique and significant advantages over other approaches such as pharmacotherapy and cognitive-behavioral therapy. A randomized crossover trial focusing on examination anxiety demonstrated that listening to calming, slow-tempo music can effectively reduce individuals’ anxiety levels and induce a state of relaxation (Lai et al., 2008). More importantly, music therapy is characterized by high social acceptability, low cost, and ease of integration into daily routines, positioning it as a highly practical alternative treatment for anxiety management (Lata and Kourtesis, 2021). These merits not only enhance the operability and adherence of the intervention but also provide a side-effect-free and accessible approach to health promotion for diverse populations. Music therapy (MT) is defined as the clinical and evidence-based use of music therapy to accomplish individualized goals within an therapeutic relationship by a credentialed professional who has completed an approved music therapy program. Such interventions are versatile in addressing diverse healthcare and educational objectives, including promoting wellness, managing stress, alleviating pain, facilitating emotional expression, enhancing memory, improving communication, and supporting physical rehabilitation, among others (Association for Music Therapy, 2005). The term music intervention is an umbrella term that refers to a range of different approaches in which music is used in a specified and systematic manner with the aim of influencing a client’s health or wellbeing… It encompasses both music therapy and music medicine, as well as other applications of music in healthcare contexts that may not fit neatly into either category (Edwards, 2016). Existing studies indicate that MT can regulate emotions and alleviate anxiety (Jia et al., 2025; Soqia et al., 2025). Moreover, MT is suitable for people with anxiety of all kinds, including college students. A single session of MT is effective in reducing anxiety and promoting relaxation, as shown by the decrease in heart rate and respiratory rate in patients receiving ventilation assistance during the intervention (Chlan, 1998). MT also has a good effect on alleviating the condition of intensive care patients (Golino et al., 2019) and shows potential in psychological intervention for college students (Xue Nan and Zhou, 2023). Furthermore, it has been found to be beneficial for anxiety types prevalent in students, such as social anxiety and test anxiety (Ying, 2011; Fan Xiaorong, 2018).

During our literature search, we identified a recent network meta-analysis by Wimbarti et al. (2025) that focuses on music listening a specific technique and compares various listening modalities. However, this meta-analysis centers primarily on discrete, technique-oriented approaches rather than examining music therapy as a holistic, integrated intervention. A comprehensive synthesis evaluating music therapy as a broad intervention category for anxiety in college students remains absent. This gap hinders the determination of its precise efficacy and limits evidence-based practical implementation (Wiljer et al., 2020). To address this, the present systematic review and meta-analysis aims to rigorously assess whether music therapy significantly reduces anxiety among college students by integrating available evidence. In doing so, we hope to offer a practical, low-cost intervention option and contribute to improving mental health literacy in this population.

## 2 Methods

### 2.1 Study registration

This meta-analysis was conducted and reported in accordance with the Preferred Reporting Items for Systematic Reviews and Meta-Analyses (PRISMA) guidelines. We attached the completed PRISMA checklist as Supplementary material and referred to the PRISMA 2020 version throughout the study (Page et al., 2021). The review protocol was prospectively registered with the International Prospective Register of Systematic Reviews (PROSPERO) (registration ID: CRD420251065591).

### 2.2 Literature search

A comprehensive systematic search was conducted across eight major electronic databases, including (Chinese: CNKI, Wanfang, VIP; English: PubMed, Web of Science, Embase, Scopus, Ovid Medline). The search included all available records from each database’s inception until December 1, 2025. A combination of free text terms and medical subject heading terms was used for the subject search. Search terms were as follows: English search terms: (“Music Therapy” OR “Therapy, Music” OR “Music” OR “Musical”) AND (“college students” OR “university students” OR “undergraduate students”) AND (“Anxiety” OR “Anxiety Disorder” OR “Disorder” OR “dysthymic disorder” OR “depressive disorder” OR “consciousness disorders” OR “Depressive” OR “Nervousness”). Chinese search term: (“yin yue liao fa” (music therapy) AND “da xue sheng” AND “jiao lv”).

### 2.3 Study selection criteria

The inclusion criteria were as follows: (1) study participants: undergraduate students (including those with anxiety symptoms); (2) intervention: MT as the primary intervention modality, including: active music therapy, passive music therapy and combined music therapy; (3) comparison: direct comparisons between different MT approaches; (4) outcomes: studies must include anxiety as an outcome measure (e.g., using validated anxiety scales); (5) study design: randomized controlled trials (RCTs).

The exclusion criteria were as follows: (1) studies involving non-university student populations; (2) interventions not incorporating MT as a primary modality, including pharmacological treatments or psychological counseling alone, or using music solely as background; (3) studies not addressing anxiety, anxiety disorders, or related psychological conditions in university students, or focusing on MT applications for physical rehabilitation or academic performance enhancement; (4) Review articles, conference abstracts, systematic reviews, meta-analyses, or other secondary literature lacking primary empirical data; (5) studies with incomplete outcome data precluding meaningful analysis; (6) repeated published studies.

### 2.4 Data extraction

Two reviewers independently screened all citations; disagreements were resolved by discussion, with arbitration by a third reviewer when consensus could not be reached. Duplicate records were removed using EndNote X9. The remaining citations were then subjected to independent full-text assessment against predefined inclusion and exclusion criteria by the same two reviewers. Data were subsequently extracted with a piloted extraction form. The extracted data included authors and year of publication, the country where the study was conducted, the type of study participants, the sample sizes of the experimental and control groups, the mean age and standard deviation of the participants, the intervention measures, the MT duration, the post-MT anxiety levels, the outcome assessment indicators, the follow-up duration, etc.

### 2.5 Quality assessment

Studies that met the inclusion criteria were evaluated for methodological quality to assess the risk of bias for each outcome. The risk of bias in trials was assessed according to the Cochrane Risk of Bias Tool (RoB 1) (Higgins et al., 2011), which evaluates seven key domains:(1) randomization sequence generation, (2) allocation concealment, (3) blinding of participants and personnel, (4) blinding of outcome assessment, (5) completeness of outcome data, (6) selective outcome reporting, (7) other potential sources of bias. Each assessment item was categorized into “low risk of bias” (yes, marked in green), “high risk of bias” (no, marked in red), or “moderate risk of bias” (unclear, marked in yellow). The responses for each item included “yes,” “no,” and “unclear,” while the overall risk of bias for each study was determined according to the highest level of bias identified across all domains. In case of disagreements among researchers during the bias classification process, they would be resolved through joint discussions between the two researchers. The results of the meta-analysis were interpreted in terms of findings regarding the risk of bias. Review Manager 5.2.3 was used to present the results graphically.

### 2.6 Statistical analysis

Statistical analyses were performed with RevMan 5.2 and Stata 16.0. When studies used different instruments to measure the same outcome, we pooled the results as standardized mean differences (SMDs) with 95 % confidence intervals (CIs). Heterogeneity across studies was assessed using the Cochran’s Q test and quantified by the I^2^statistic. According to the thresholds proposed by Higgins and Thompson (2002), I^2^ values of approximately 25, 50, and 75% are interpreted as low, moderate, and high heterogeneity, respectively. All analyses were performed using a random-effects model throughout the study. To explore sources of heterogeneity, we conducted pre-specified subgroup analyses and sensitivity analyses. Publication bias was evaluated by visual inspection of funnel plots and formally tested with the Egger’s regression. If the funnel plot showed an asymmetric distribution and the *P*-value of Egger’s test was < 0.05, publication bias was considered to exist.

## 3 Results

### 3.1 Literature screening process

**FIGURE 1:**
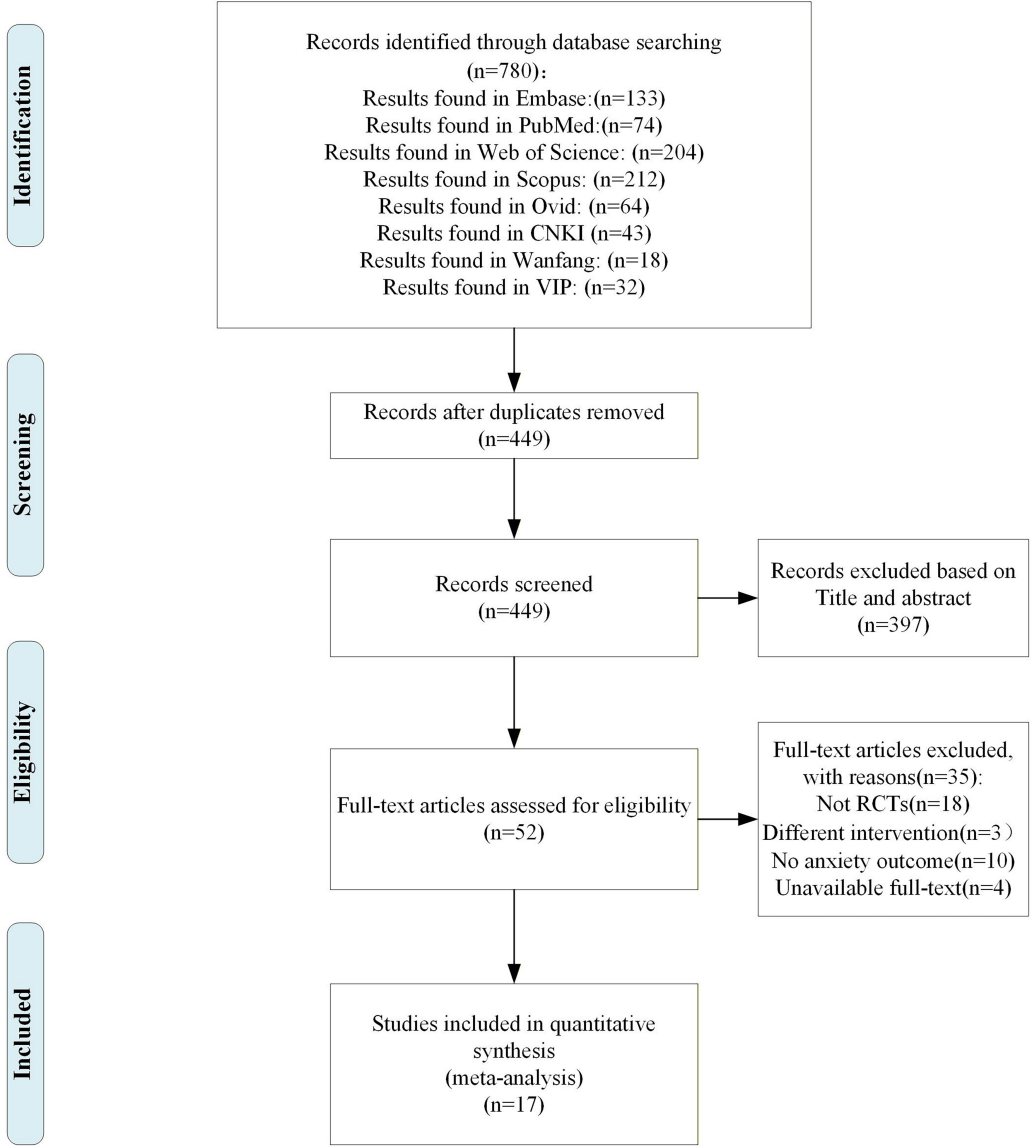
Literature screening process.

The initial search identified 780 records. Following the removal of 331 duplicates, 449 unique citations were retained. Title and abstract screening excluded 397 studies that failed to satisfy preliminary criteria. A full-text review of the remaining 52 articles led to the exclusion of 35 reports, primarily due to failure to satisfy predefined inclusion requirements. Consequently, 17 studies (including 18 RCTs) qualified for inclusion in the final analysis (WU Wei, 2009; Yinghua and Yi, 2017; Gong Guangjun and Zhou, 2019; Lee and Bae, 2020; Jin, 2023; Hong, 2024; Qi et al., 2024; Vasylevska-Skupa et al., 2024; Xu Sun et al., 2024; Yao et al., 2024; Liu et al., 2025; Wang et al., 2025). The flowchart of literature selection was shown in Figure 1.

### 3.2 Characteristics of included studies

A summary of the basic characteristics of included studies was presented in Table 1. As shown in Table 1. A total of 17 studies involving 11,679 participants were finally included in this meta-analysis. The studies were published between 2009 and 2025, with sample sizes ranging from 24 to 378 participants. The majority of the studies were from China (WU Wei, 2009; Aixue Sun and Shuying, 2012; Yinghua and Yi, 2017; Gong Guangjun and Zhou, 2019; Rong, 2022; Jin, 2023; Hong, 2024; Qi et al., 2024; Xu Sun et al., 2024; Yao et al., 2024; Yingying, 2024; Liu et al., 2025; Wang et al., 2025; Xu et al., 2025), the remaining three were conducted in Korea, England, and Ukraine (Elliott et al., 2014; Lee and Bae, 2020; Vasylevska-Skupa et al., 2024). Three studies reported the time of follow-up (Rong, 2022; Hong, 2024; Qi et al., 2024).

**TABLE 1:** The basic characteristics of the eligible studies.

Athor, year	Country	Type of participants	Sample size (E/C)	Age in years (M ± SD)	Measurement tools used in each eligible study	Intervention	Types of MT	Intervention frequency	Length of MT sessions	Control	Outcome measures	Music style	Interference transmission mode	Recruitment mode	Categories of anxious people	Length of follow-up
Rong (2022)	China	College student athlete	25/24	E:(21.57 ± 5.39) C:(21.57 ± 5.39)	Sports competition anxiety questionnaire	MT	Combination	Regularly every week	7 weeks	UC	Five-Dimensional Mindfulness Questionnaire. Sports competition anxiety questionnaire	Comprehensive MT	Offline	Offline recruitment	Sports competition anxiety/training stress-related anxiety	3 months
Lee and Bae (2020)	Korea	College students with anxiety	31/31	E:(22.77 ± 2.43) C:(22.13 ± 1.66)	NRS	MT	Passive	Single intervention	Once every 30 min	UC	Stress Index EEG HR NRS	Modern music	Offline	Offline recruitment	Universal anxiety	None
Elliott et al. (2014)	England	College students with anxiety	26/26	E:(19.42 ± 1.9) C:(19.42 ± 1.9)	CSAI-2R	MT	Passive	Single intervention	10 min each time	UC	CSAI-2R Subjective relaxation score HR Performance in sports tasks	Western modern music	Offline	Voluntary registration	Anxiety of competition state	None
Qi et al. (2024)	China	College students with anxiety	12/12	E:(20.83 ± 1.267) C:(20.83 ± 1.267)	Self-rated anxiety questionnaire	MT	Combination	Single intervention	45 min each time	UC	Self-reported Anxiety Questionnaire Open-ended subjective feelings survey	Modern music	Offline	Combination of online and offline/voluntary registration	Universal anxiety	None
Vasylevska-Skupa et al. (2024)	Ukraine	College students majoring in music	95/95	E:(20.26 ± 1.46) C:(20.26 ± 1.46)	K-MPAI	MT	Active	Once a week	one academic year	UC	K-MPAI SF-36 UCLA Loneliness CISS CD-RISC	Vocal MT	Offline	Offline recruitment	performance anxiety/academic stress-related anxiety	None
Xu et al. (2025)	China	College students with anxiety	60/60	E:(20.7 ± 2.2) C:(20.3 ± 2.4)	SAS	MT	Passive	Twice a week	12 weeks	UC	SDS SAS	Western classical music	Offline	Online + offline	Universal anxiety	None
Liu et al. (2025)	China	Senior undergraduate student	90/90	E:(22.63 ± 0.23) C:(22.63 ± 0.23)	TAS	MT	Combination	Twice a week 60 min each time	7 months	UC	WEMWBS 11TAS	Mixed music genre	Offline	Combination of online and offline	Academic stress-related anxiety	None
Jin (2023),	China	College students with mild to moderate depressive and anxious symptoms	13/13	E:(20.04 ± 1.65) C:(20.4 ± 1.65)	SCL-90	MT	Passive	Once a week 60 min each time	8 weeks	UC	SCL-90 SDS SAS	Post-rock music	Online + offline	Online + offline/voluntary registration	Universal anxiety	8 weeks
Yao et al. (2024)	China	A sophomore student from a higher vocational medical college	37/37	E:(19.37 ± 0.1) C:(19.33 ± 0.66)	SAS	MT	passive	Once a day 15 min each time	28 days	UC	SDS SAS PSQI	Oriental music	Offline	Offline+ Voluntary participation	Academic stress-related anxiety/Depression is associated with anxiety	None
WU Wei (2009)	China	College students with post-traumatic anxiety symptoms after the earthquake	15/15	E:(20 ± 2) C:(20 ± 2)	SAS	MT	Combination	Regular psychological counseling	1 month	TAU	SAS	Western classical music	Offline	Offline+ random allocation	Post-traumatic anxiety/Situational anxiety/comorbid anxiety	None
Hong (2024)	China	College students with test anxiety	15/15	E:(19.6 ± 2) C:(19.6 ± 2.5)	TAI	MT	Combination	Twice a week	4 weeks	UC	TAI SSS	Mixed type	Offline+ on-line	Offline+ Voluntary participation	Examination anxiety/Somatic anxiety/Academic stress-related anxiety	2 months
Yingying (2024)	China	College students with social anxiety and sleep problems	60/60	E:(21.38 ± 1.23) C:(21.64 ± 1.45)	SCL-90	MT	Combination	Once or twice a week	2 years	UC	SAD SCL-90 PSQI SF-36 Simplify the comfort status scale	Mixed type	Offline+ on-line	Offline+ Voluntarily participate and meet the inclusion criteria	Social anxiety/Academic stress-related anxiety/Anxiety is accompanied by sleep disorders	None
Aixue Sun and Shuying (2012)	China	College students preparing for the postgraduate entrance examination	55/62	E:(21.58 ± 1.3) C:(21.53 ± 1.25)	TAI	MT	Passive	Once a week 20 min each time	6 weeks	UC	TAI	Western relaxation music	Offline	Offline recruitment	Examination anxiety/Situational anxiety	None
Yinghua and Yi (2017)	China	College students with first-episode depression	30/30	E:(22.64 ± 2.82) C;(23.45 ± 2.29)	SAS	MT	Passive	Once a week	12 weeks	TAU	SAS SDS GQOLI-74	Oriental music	Offline	Offline	Depression is associated with anxiety/pathological anxiety	None
Gong Guangjun and Zhou (2019)	China	Junior college students in their second or third year who are facing employment	42/42	E:(22.5 ± 2.2) C:(23 ± 2.17)	Employment anxiety scale for college graduates	MT	Passive	Once a week	1 month	UC	Core scale	Mixed type	Offline	Offline+Voluntary participation	Employment anxiety+	None
Xu Sun et al. (2024)	China	College students with anxiety in medical colleges and universities	27/26	E;(20.25 ± 2.13) C:(20.08 ± 2.077)	SAS	MT	Passive	Twice a week	6 weeks	UC	SAS SCL-90 Sleep Quality Self-assessment form	Oriental music	Offline	Offline+Voluntary participation	Universal anxiety/somatic anxiety/traditional Chinese medicine syndrome differentiation types of anxiety	None
Wang et al. (2025)	China	Senior college students (enrolled in Music Appreciation elective course)	181/197	E: (20.16 ± 0.73); C: (20.13 ± 0.74)	SAS	Mindfulness-based music therapy (MBMI)	Combination	Once a week (40 min each time) + 15–20 min daily homework	6 weeks	UC	SAS, SDS, OSS, PSQI, CD-RISC	Classical music + personalized selected music	Offline	Offline recruitment+ random allocation	Academic stress-related anxiety + postgraduation anxiety	None

E, Experimental group; C, Control group; M, Mean; SD, Standard deviation; MT, MT; TAU, Treatment as usual; UC, Usual care; EEG, Electroencephalogram; HR, Heart Rate; NRS, Numerical Rating Scale; CSAI-2R,Competition State Anxiety Inventory-2 Revised; K-MPAI, Kenny Music Performance Anxiety Inventory; SF-36,36-Item Short-Form Health Survey; UCLA Loneliness, University of California, Los Angeles Loneliness Scale; CISS, Coping Inventory for Stressful Situations-Short Form; CD-RISC, Connor-Davidson Resilience Scale; SDS, The Self-Rating Depression Scale; SAS, The Self-Rating Anxiety Scale; WEMWBS, Warwick-Edinburgh Mental Wellbeing Scale; 11TAS,Trait Anxiety Scale, 11-item version; SCL-90,Symptom Checklist-90;PSQI, Pittsburgh Sleep Quality Index; TAI, Test Anxiety Inventory; SSS, Somatization Symptom Scale; SAD, Social Avoidance and Distress Scale; GQOLI-74,Generic Quality of Life Inventory-74;Length of follow-up, the time between the end of the intervention and the outcome measure of follow-up in the included study; None, Not mention.

### 3.3 Risk of bias

**FIGURE 2:**
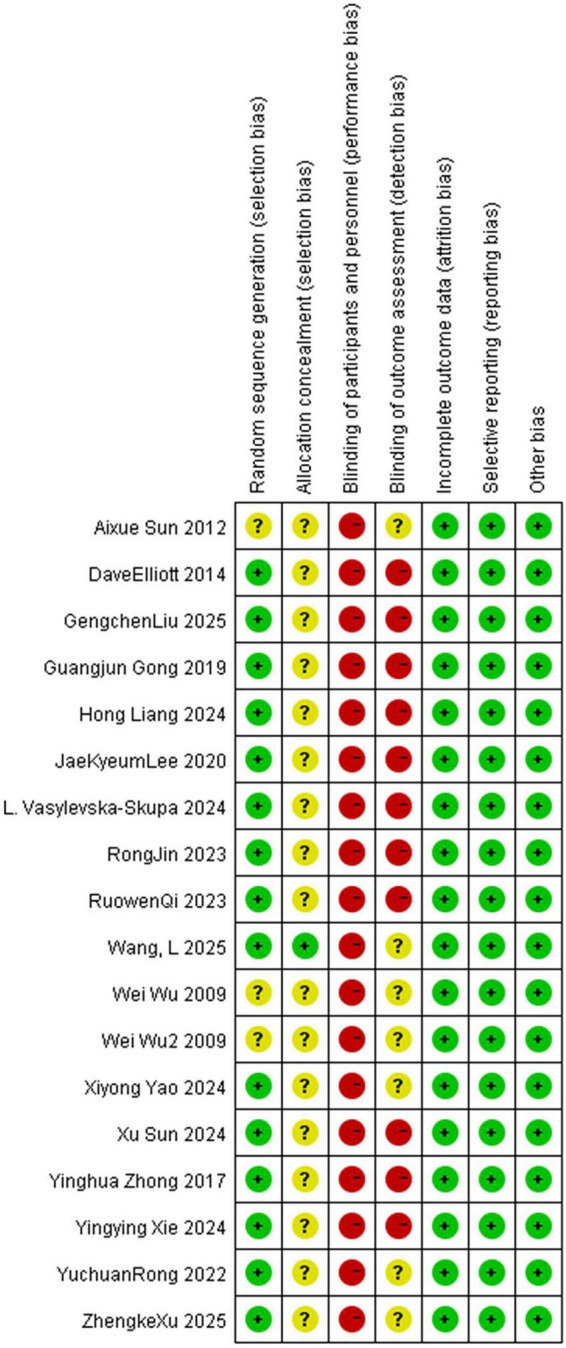
Risk of bias assessment for included studies. It evaluates each study across multiple bias domains: random sequence generation (selection bias), allocation concealment (selection bias), blinding of participants and personnel (performance bias), blinding of outcome assessment (detection bias), incomplete outcome data (attrition bias), selective reporting (reporting bias), and other bias. Green indicates low risk of bias, blank indicates unclear risk of bias, and red indicates high risk of bias.

The results of the risk of bias assessment were presented in Figure 2. Regarding random sequence generation, 15 studies were rated as “low risk of bias” (Elliott et al., 2014; Yinghua and Yi, 2017; Gong Guangjun and Zhou, 2019; Lee and Bae, 2020; Rong, 2022; Jin, 2023; Hong, 2024; Qi et al., 2024; Vasylevska-Skupa et al., 2024; Xu Sun et al., 2024; Yao et al., 2024; Yingying, 2024; Liu et al., 2025; Wang et al., 2025; Xu et al., 2025), while two studies did not provide sufficient details (WU Wei, 2009; Aixue Sun and Shuying, 2012). Notably, 16 studies failed to adequately describe the implementation of allocation concealment, resulting in an unclear risk in this domain (WU Wei, 2009; Aixue Sun and Shuying, 2012; Elliott et al., 2014; Yinghua and Yi, 2017; Gong Guangjun and Zhou, 2019; Lee and Bae, 2020; Rong, 2022; Jin, 2023; Hong, 2024; Qi et al., 2024; Vasylevska-Skupa et al., 2024; Xu Sun et al., 2024; Yao et al., 2024; Yingying, 2024; Liu et al., 2025; Xu et al., 2025). In terms of blinding, all 17 studies were judged to have a “high risk of bias” in participant blinding, with 11 studies also exhibiting a “high risk of bias” in outcome assessor blinding (Elliott et al., 2014; Yinghua and Yi, 2017; Gong Guangjun and Zhou, 2019; Lee and Bae, 2020; Jin, 2023; Hong, 2024; Qi et al., 2024; Vasylevska-Skupa et al., 2024; Xu Sun et al., 2024; Yingying, 2024; Liu et al., 2025). However, all included studies were rated as having a low risk of bias across the three domains: incomplete outcome data, selective outcome reporting, and other potential sources of bias, with no significant attrition, missing data, or other potential bias issues identified (WU Wei, 2009; Aixue Sun and Shuying, 2012; Elliott et al., 2014; Yinghua and Yi, 2017; Gong Guangjun and Zhou, 2019; Lee and Bae, 2020; Jin, 2023; Hong, 2024; Qi et al., 2024; Vasylevska-Skupa et al., 2024; Xu Sun et al., 2024; Yao et al., 2024; Yingying, 2024; Liu et al., 2025; Wang et al., 2025; Xu et al., 2025).

### 3.4 Meta-analysis of MT on anxiety reduction among college students

**FIGURE 3:**
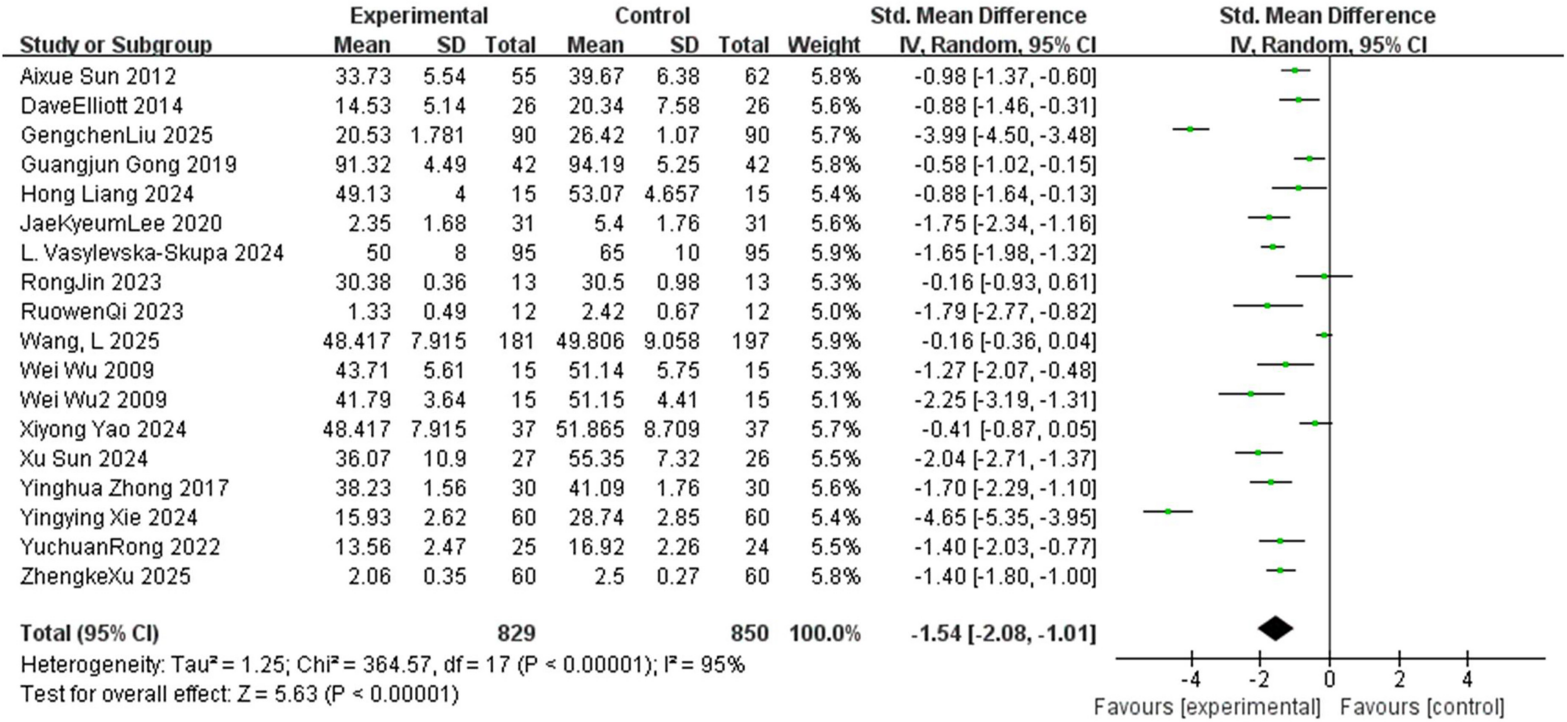
Forest plot showing meta-analysis of MT on anxiety reduction in college students. Each line represents an individual study, presenting its standardized mean difference and 95% confidence interval (CI). Negative values indicate that the effect favors the control group, while positive values indicate that the effect favors the music therapy (experimental) group. The size of the squares is proportional to the weight of each study. The diamond represents the pooled effect size under a random-effects model, with its width corresponding to the 95% CI. The vertical line at zero is the line of no effect.

The sixteen included studies involved 1,679 participants, with 829 from the experimental group and 850 from the control group. As shown in Figure 3, significant heterogeneity was observed across studies (*I*^2^ = 95%, *P* < 0.001), indicating that 95% of the variability in results stemmed from true differences between studies (rather than sampling error). A random-effects model was therefore used to account for this substantial between-study variability. The meta-analysis demonstrated that MT significantly reduced anxiety symptoms compared to control groups (SMD = –1.54, 95% CI: –2.08 to –1.01, *P* < 0.05), accompanied by high heterogeneity (*I*^2^ = 95%). However, the high heterogeneity (*I*^2^ = 95%) limits the interpretability of this pooled effect size—it should not be generalized as a uniform effect of MT. Instead, subsequent subgroup analyses (stratified by MT type, length of MT sessions, and different countries) were conducted to explore the sources of heterogeneity and identify specific contexts in which MT is most effective.

### 3.5 Subgroup analyses

#### 3.5.1 Meta-analysis of anxiety reduction among college students with different types of MT

**FIGURE 4:**
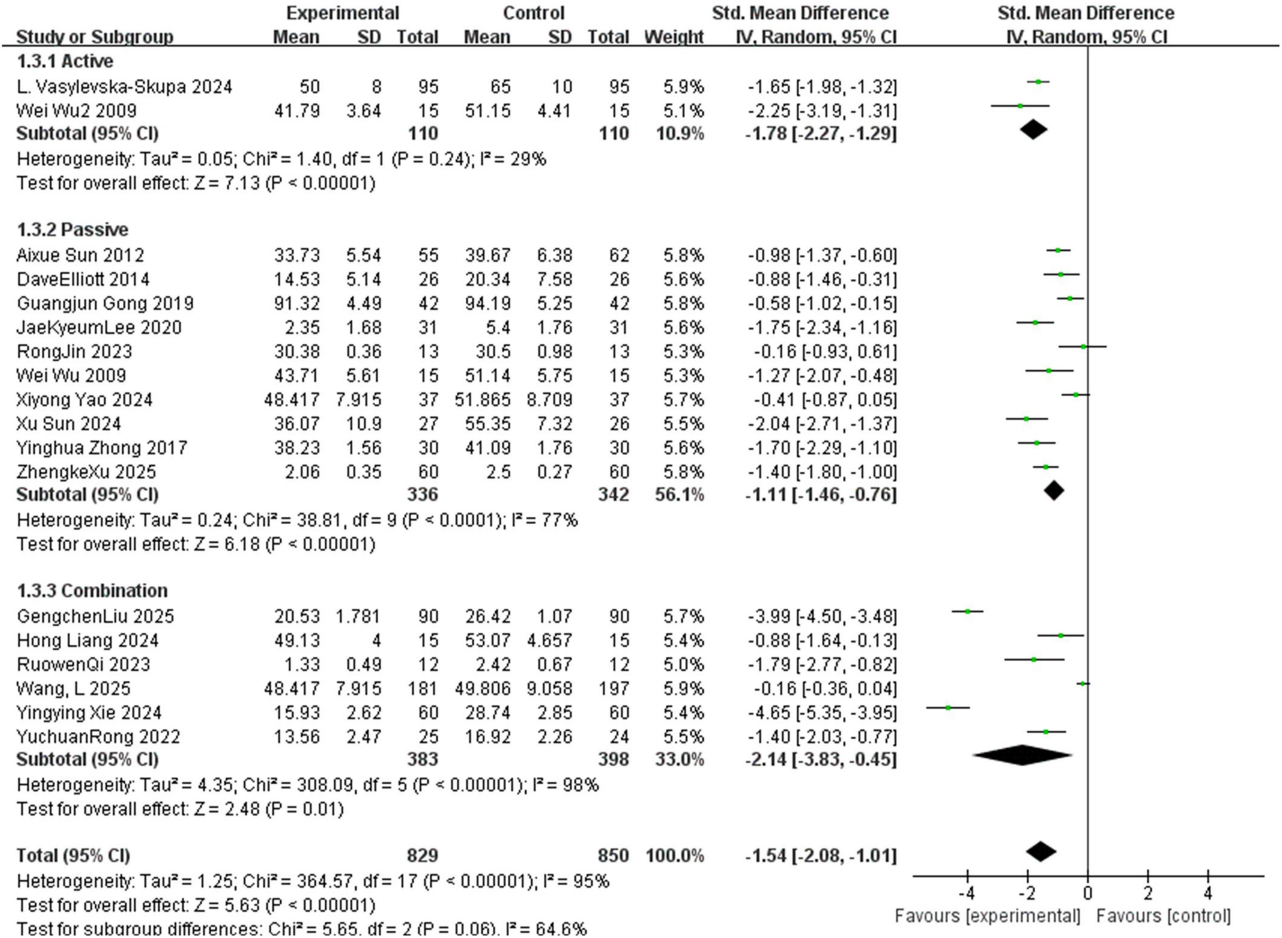
Forest plot for meta-analysis of anxiety reduction among college students with different types of MT (music therapy), showing effects of active, passive, and combination music interventions on anxiety between experimental (music—receiving) and control groups. Each line represents a study, with standardized mean difference and 95% CI (negative = control-favorable, positive = experimental-favorable). Square size matches study weight; the diamond is pooled effect (random-effects model) with 95% CI width. Vertical zero line = no-effect line. Subgroups: “Active,” “Passive,” “Combination,” “Total” aggregates all studies.

Eighteen randomized controlled trials (total sample size *N* = 1,679) specifically explored the impact of three distinct types of music therapy (MT) on outcome measures, and these studies were divided into the following three subgroups: active (*n* = 2) (WU Wei, 2009; Vasylevska-Skupa et al., 2024), combined (*n* = 6) (Rong, 2022; Hong, 2024; Qi et al., 2024; Yingying, 2024; Liu et al., 2025; Wang et al., 2025), and passive (*n* = 10) (WU Wei, 2009; Aixue Sun and Shuying, 2012; Elliott et al., 2014; Yinghua and Yi, 2017; Gong Guangjun and Zhou, 2019; Lee and Bae, 2020; Jin, 2023; Xu Sun et al., 2024; Yao et al., 2024; Xu et al., 2025). The active MT subgroup significantly improved outcomes compared with the control group (SMD = –1.78, 95%CI: –2.27 to –1.29, *p* > 0.05, *I*^2^ = 29%) with low heterogeneity and reliable results; the passive MT subgroup (SMD = –1.11, 95%CI: –1.46 to –0.76, *p* < 0.05, *I*^2^ = 77%) with high heterogeneity; and the combined MT subgroup, which had the largest effect size (SMD = -2.14, 95%CI: -3.83 to -0.45, *p* < 0.05, *I*^2^ = 98%) but extremely high heterogeneity. Test for subgroup differences indicated no statistically significant difference in effect sizes among the three subgroups (*p* > 0.05, *I*^2^ = 64.6%) (Figure 4).

#### 3.5.2 Meta-analysis of anxiety reduction among college students in different countries

**FIGURE 5:**
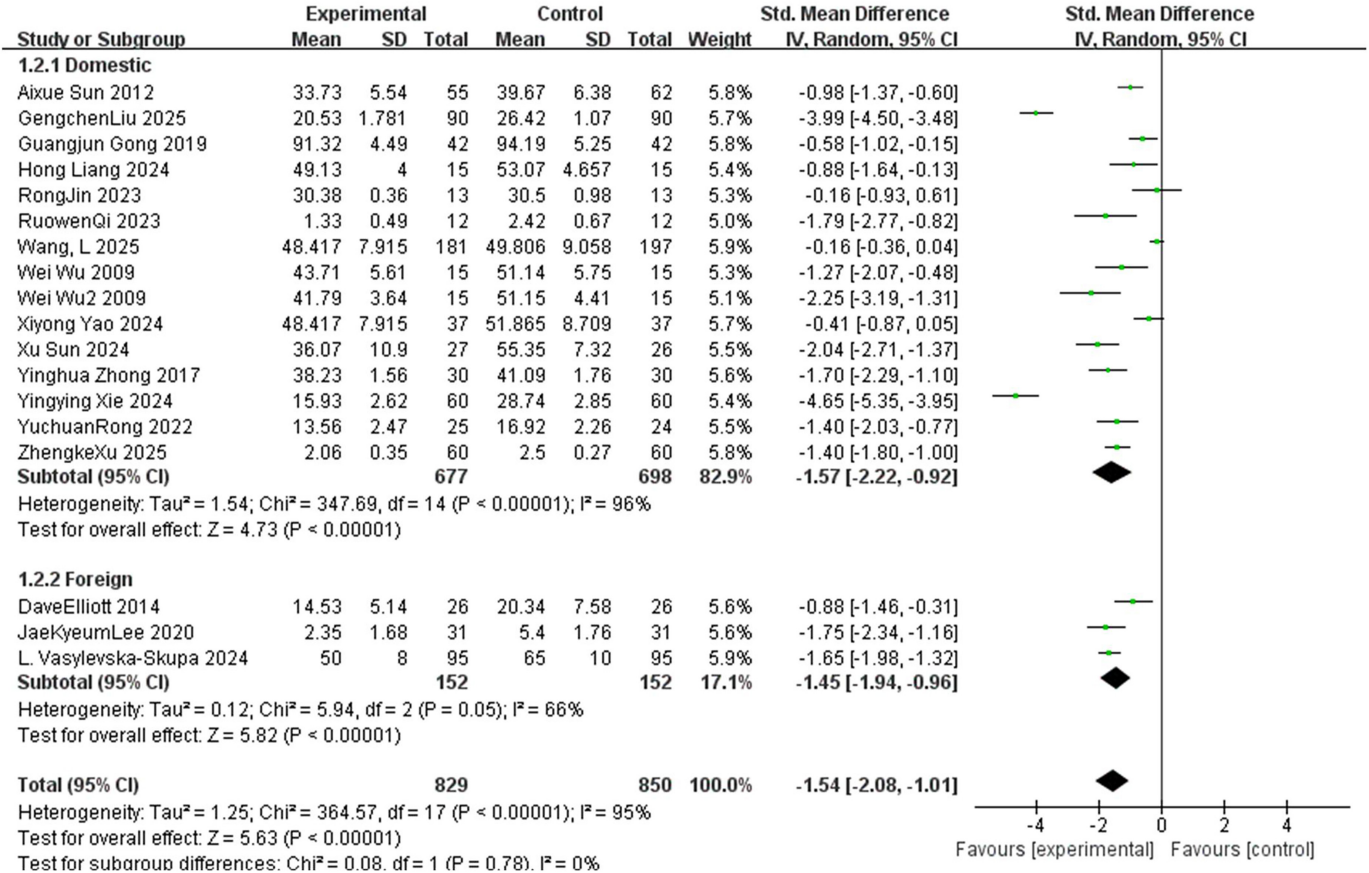
Forest plot for meta-analysis of anxiety reduction among college students in different countries, showing the effect of interventions on anxiety between experimental and control groups. Each line represents a study, with standardized mean difference and 95% CI (negative values favor control, positive favor experimental). Square size corresponds to study weight; the diamond is the pooled effect (random-effects model) with 95% CI width. The vertical zero line is the no-effect line. Subgroups: “Domestic” and “Foreign”; “Total” aggregates all studies.

Seventeen included studies examined the efficacy of MT for anxiety reduction across different countries. For subgroup analysis, studies were categorized as domestic (*n* = 14) (WU Wei, 2009; Aixue Sun and Shuying, 2012; Yinghua and Yi, 2017; Gong Guangjun and Zhou, 2019; Rong, 2022; Jin, 2023; Hong, 2024; Qi et al., 2024; Xu Sun et al., 2024; Yao et al., 2024; Yingying, 2024; Liu et al., 2025; Wang et al., 2025; Xu et al., 2025) or international (*n* = 3) (Elliott et al., 2014; Lee and Bae, 2020; Vasylevska-Skupa et al., 2024). The meta-analysis showed that domestic studies demonstrated a statistically significant anxiolytic effect of MT compared to controls (SMD = -1.57, 95%CI: -2.22 to -0.92, *p* < 0.05, *I*^2^ = 96%), while foreign studies also showed a significant improvement effect (SMD = -1.45, 95%CI: -1.94 to -0.96, *p* = 0.05, *I*^2^ = 66%) (Figure 5). Given limited international studies (*n* = 3), we cannot reliably determine whether MT efficacy differs across cultures.

#### 3.5.3 Meta-analysis of anxiety reduction among college student with different length of MT sessions

**FIGURE 6:**
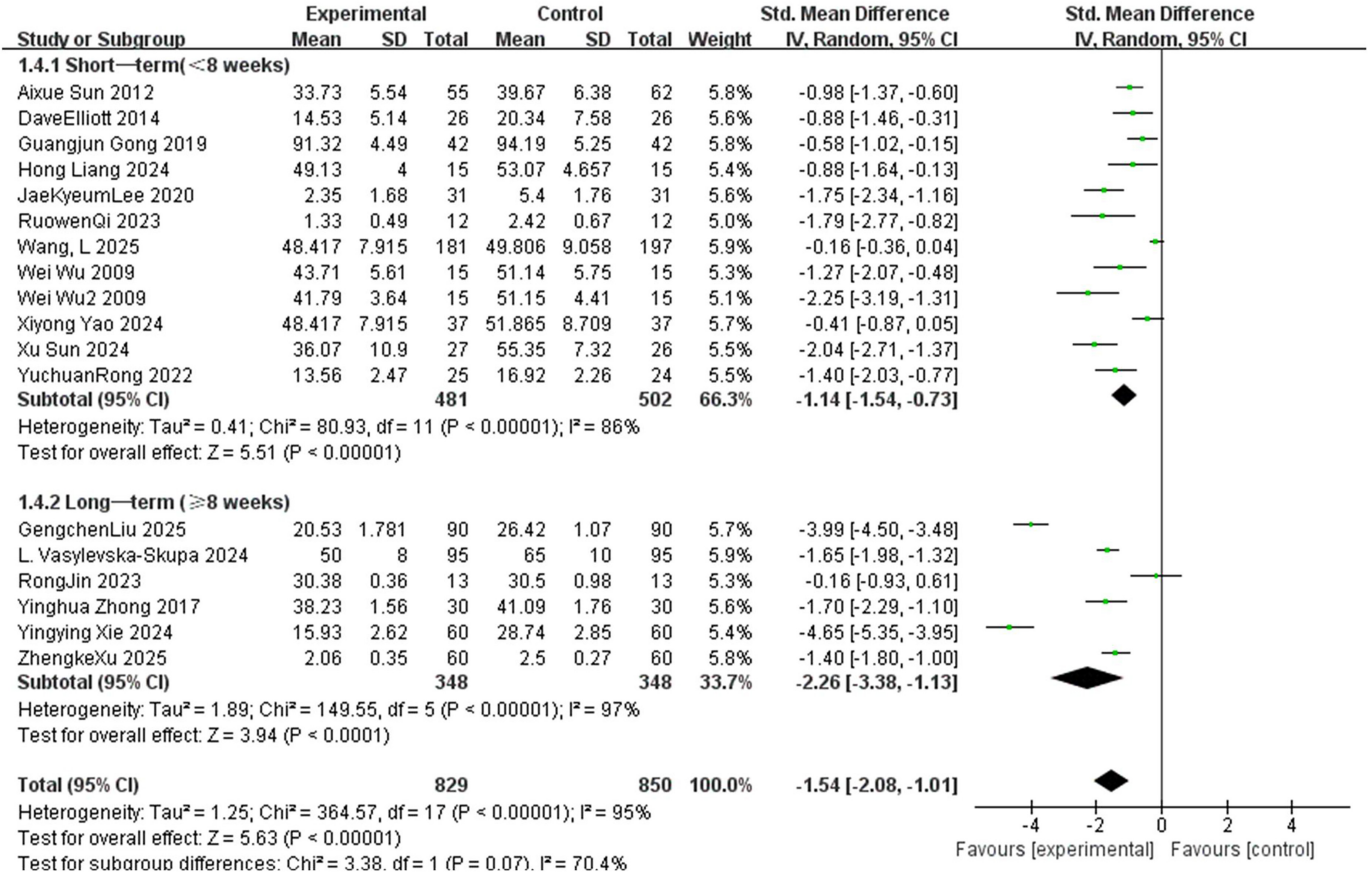
Forest plot for meta-analysis of anxiety reduction among college students with different length of MT sessions, showing the effect of interventions on anxiety between experimental and control groups. Each line represents a study, with standardized mean difference and 95% CI (negative values favor control, positive favor experimental). Square size corresponds to study weight; the diamond is the pooled effect (random-effects model) with 95% CI width. The vertical zero line is the no-effect line. Subgroups: “Short-term intervention ( < 8 weeks)” and “Long-term intervention ( ≥ 8 weeks)”; “Total” aggregates all studies.

Seventeen included studies evaluated the effects of MT on anxiety with varying length of MT sessions. These studies were divided into two groups for subgroup analysis: short-term MT ( < 8 weeks, *n* = 11) (WU Wei, 2009; Aixue Sun and Shuying, 2012; Elliott et al., 2014; Gong Guangjun and Zhou, 2019; Lee and Bae, 2020; Rong, 2022; Hong, 2024; Qi et al., 2024; Xu Sun et al., 2024; Yao et al., 2024; Wang et al., 2025) and long-term MT ( ≥ 8 weeks, *n* = 6) (Yinghua and Yi, 2017; Jin, 2023; Vasylevska-Skupa et al., 2024; Yingying, 2024; Liu et al., 2025; Xu et al., 2025). In this study, the length of MT sessions was divided into short-term ( < 8 weeks) and long-term ( ≥ 8 weeks) for subgroup analysis, with 8 weeks as the cutoff point. The cutoff point was set with consideration for both the completeness and feasibility of the intervention protocol, as an approximately 8-week intervention was deemed a critical period to assess the efficacy of music therapy. For example, in a cognitive rehabilitation study on multiple sclerosis, Aalbers et al. (2017) adopted an 8-week comprehensive neurologic music therapy protocol, which confirmed that this duration could accommodate a structured treatment unit and allow observation of significant changes in therapeutic effects (Aalbers et al., 2017). Additionally, this cutoff point was consistent with the common short-term MT cycles (6–12 weeks) in international clinical psychology research (Impellizzeri et al., 2020) and aligned with the natural distribution of length of MT sessions among the studies included in this meta-analysis, ensuring the comparability of sample sizes between subgroups. The subgroup meta-analysis indicated that MT demonstrated a significant positive effect on anxiety compared to the control group. Specifically, the short-term MT subgroup (16 studies, weight 66.3%) showed a statistically significant MT effect (SMD = -1.14, 95%CI: -1.54 to -0.73, *p* < 0.05, *I*^2^ = 86%); the long-term MT subgroup (7 studies, weight 33.7%) not only had a significant improvement effect but also a higher effect size (SMD = –2.26, 95%CI: –3.38 to –1.13, *p* < 0.05, *I*^2^ = 97%) (Figure 6).

### 3.6 Sensitivity analysis and publication bias

**FIGURE 7:**
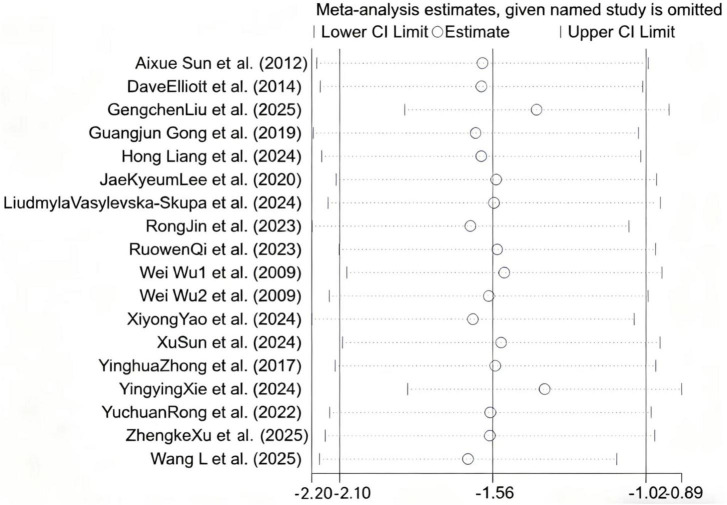
Sensitivity analysis plot for the meta-analysis. It shows meta-analysis estimates when each named study is omitted one by one. The vertical line represents the overall effect estimate of the meta-analysis when all studies are included. Each circle denotes the effect estimate after omitting a specific study, and the horizontal lines extending from the circle indicate the 95% confidence interval (CI) for that estimate.

**FIGURE 8:**
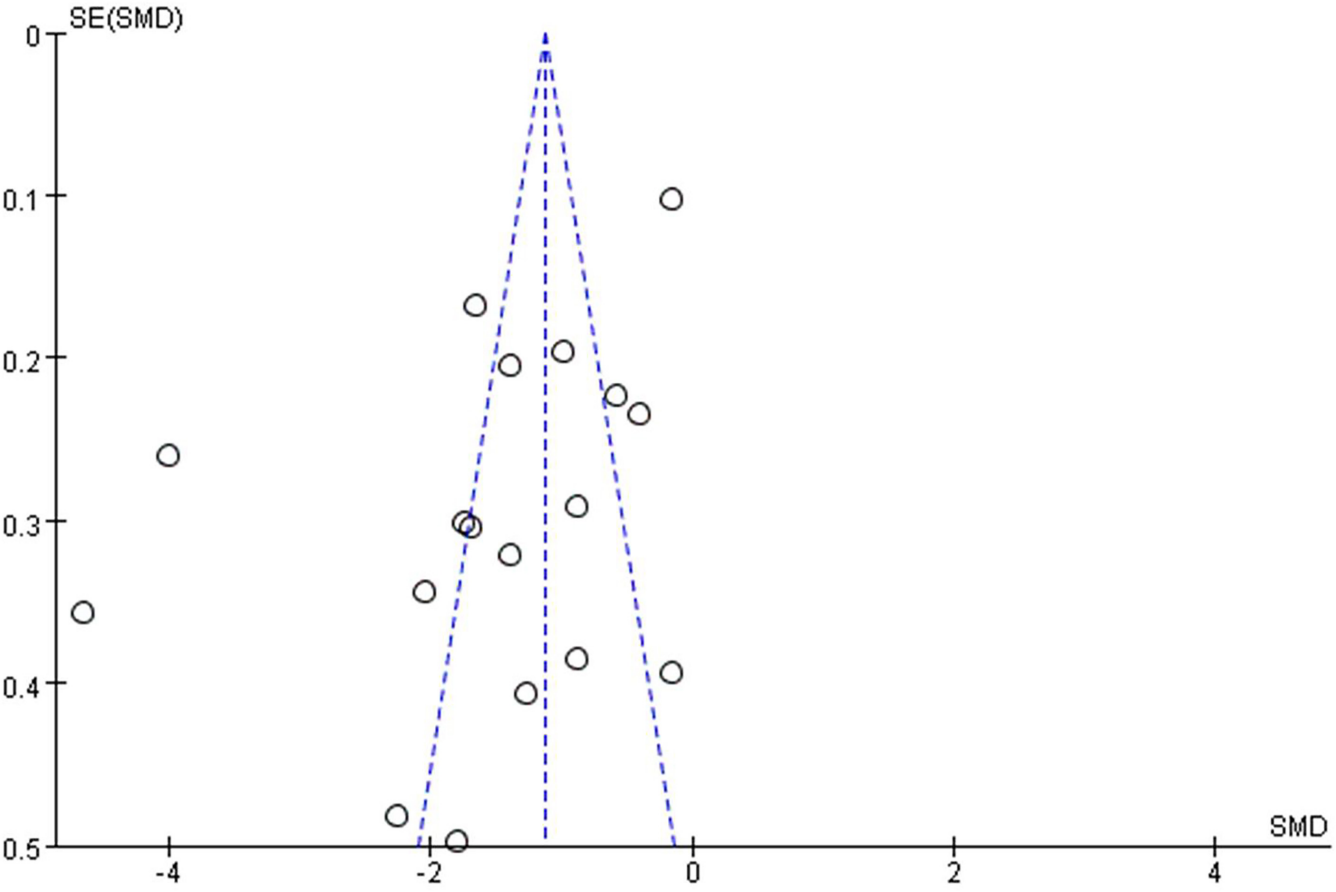
Funnel plot of included studies.

To evaluate the robustness of the study findings, a sensitivity analysis was performed using the exclusion method for literatures with high heterogeneity. The results showed that while the magnitude of the pooled effect size exhibited modest fluctuations (range: –2.51 to –0.95) after excluding any single study, the outcome remained statistically significant—the 95% CI never crossed zero (Figure 7). Therefore, these findings provided evidence that MT is effective in mitigating anxiety. To assess the possibility of publication bias, we conducted Egger’s linear regression test and visual inspection of the funnel plot. The 18 included studies showed a certain degree of symmetry in the funnel plot, with most clustered in the low-middle section and a relatively balanced distribution on both sides (Figure 8). Egger’s test was performed to assess publication bias among the 17 included RCTs. The results indicated the presence of statistically significant publication bias (*P* = 0.025 < 0.05). Further combined with the results of sensitivity analysis, Egger’s test was performed after sequentially excluding each single study. It was found that after excluding one large-sample study (Wang et al., 2025), the *P*-value of Egger’s test changed from 0.025 to 0.472, failing to reject the null hypothesis of “no publication bias,” which suggests that this large-sample study is the key dominant factor contributing to the overall publication bias.

## 4 Discussion

This meta-analysis combined 18 randomized controlled trials that involved a total of 1,679 college students. The results indicated that, compared to the control group, Music therapy (MT) significantly alleviated anxiety symptoms among college students. The mechanisms of action might involve the following aspects. Firstly, as a multidimensional composite stimulus, the unique structure (e.g., melody, rhythm), emotional attributes, and often accompanying cultural and personal memories of music enabled it to capture and sustain an individual’s attention more effectively than general pleasant stimuli. This effectively redirected focus from anxiety-inducing sources to positive perceptual experiences (Kasuya-Ueba et al., 2020; Woods et al., 2024). Secondly, MT was likely to inhibit the activity of the hypothalamic-pituitary-adrenal (HPA) axis, reducing the secretion of the stress hormone cortisol, and thereby alleviating physiological stress responses such as increased heart rate and blood pressure (McKinney et al., 1997; Thoma et al., 2013). Furthermore, it was important to recognize that the mechanisms of MT were not uniform and were influenced by individual differences (e.g., musical training, personal preference) and MT types (McPherson et al., 2019; Ab Shukor et al., 2023). For instance, receptive listening might primarily work through emotional disclosure and autonomic regulation, while active engagement (e.g., drumming or singing) could operate by enhancing agency, social connection, and entrainment of physiological rhythms (Kokal et al., 2011; Perkins et al., 2016). Future research is necessary to further elucidate these specific pathways and the interactions between individual characteristics and MT modalities. Additionally, our findings aligned with the neurobiological model that posits MT’s efficacy in alleviating anxiety through the activation of the brain’s reward circuit and the subsequent release of neurotransmitters, such as dopamine, thereby fostering positive emotional experiences and establishing a physiologically-psychological virtuous cycle (Lu et al., 2021). This mechanism was corroborated by a recent systematic review by Yan et al. (2025), which specifically focused on perioperative ophthalmic patients and demonstrated a significant anxiety-reducing effect of MT, with reported effect sizes falling within the moderate to large range. While our results were consistent with this broader trend of MT’s efficacy, the present meta-analysis provided distinct incremental value: it quantitatively synthesized evidence from a homogeneous population of college students, a group facing unique developmental and academic stressors, and confirmed that the moderate-to-large beneficial effects (as indicated by our pooled effect size) were generalizable to this non-clinical yet high-anxiety context. Therefore, our work extended the findings from clinical settings (e.g., perioperative care) to the domain of preventive and developmental mental health support in educational environments. However, most of the previous studies only examined the effects of MT on anxiety in other populations (Blood and Zatorre, 2001; White, 2001; Sharda et al., 2019; Niu and You, 2023), with limited attention to this vulnerable group in universities. This meta-analysis specifically explored the effect of MT on the anxiety among college students, aiming to further confirm the role of MT in alleviating anxiety and provide an effective solution that was culturally and campus-scene adapted for addressing the anxiety problem among Chinese college students.

### 4.1 Subgroup-specific effects

The subgroup analysis results indicated that there were significant differences in the intervention effects of different MT modes. Relevant studies have showed that MT is an important means to enhance an individual’s sense of happiness and self-efficacy. It comes in various forms, including active, passive and receptive MT, and these therapies were beneficial to both the physical and mental aspects (Heiderscheit et al., 2011; O’Callaghan et al., 2014). In this meta-analysis, it was found that the effect scale of combined MT (active + passive) was superior to that of the simple passive and active MT methods. The efficacy of combined music therapy (MT) could be attributed to its integration of multiple interventional pathways, including cognitive engagement (e.g., music creation) and emotional regulation (e.g., guided listening) (de Witte et al., 2022). In the current synthesis, this aligned with the finding that passive MT effectively reduced anxiety among college students (Yuhua and Yanqing, 2007; Aixue Sun and Shuying, 2012): The pooled standardized mean difference (SMD) was -1.11 (95% confidence interval [CI]: -1.46 to -0.76) across 10 included studies (*P* < 0.00001), which confirmed the favorable effect of passive MT. However, the effect of active MT was not statistically significant in this synthesis: the pooled SMD was –0.45 (95% CI: –2.27 to 1.29) based on two included studies (total sample size *n* = 201, *P* = 0.24). Since this conclusion relied on a small number of studies, caution was warranted when interpreting it. The non-significant result might not reflect a definitive lack of efficacy for active MT but could stem from several factors: (1) insufficient statistical power due to the limited number of studies and small total sample size; (2) methodological specifics of the included trials (e.g., intervention protocols, dosage, or facilitator competence); or (3) participant characteristics in these studies, such as baseline anxiety levels or musical background. Thus, this result should be regarded as inconclusive rather than indicative of ineffectiveness, which highlighted a critical research gap—future studies needed to explore the potential of active MT in this population through higher-quality, larger-scale trials. In addition, different characteristics of the participants also affected the MT effect. Students faced a variety of sources of anxiety, including academic, employment, and other pressures, which often led to internal competition anxiety, psychological imbalance, and a predisposition to anxiety, unease, depression, and other symptoms (Zheng and Peng, 2025). These factors also impaired the efficacy of MT. Therefore, it is necessary to provide personalized interventions to further validate these results in the future.

In addition, subgroup analysis by country revealed a more significant effect size in domestic studies (*n* = 14) compared to international studies (*n* = 3), which did not show statistically significant effects (Kanekar et al., 2009). However, this observed discrepancy should be interpreted with considerable caution. The most plausible explanation is the limited number and potentially smaller sample sizes of the available international studies, resulting in insufficient statistical power to detect a significant effect. Beyond this, several other methodological factors could account for the difference. These may include variations in the specific MT protocols employed, differences in the choice and rigor of control conditions, or the use of culturally adapted anxiety assessment tools that may not be directly comparable. Furthermore, the possibility of publication bias, wherein positive results from the domestic context are more readily published, cannot be ruled out. Therefore, attributing the difference solely to contextual factors like academic pressure is premature. Future research should prioritize high-quality, multi-national studies with standardized protocols to clarify the role of cultural and contextual moderators in MT’s efficacy for anxiety.

In this study, the subgroup analysis regarding the duration of MT revealed that MT was able to significantly alleviate the anxiety of college students, regardless of whether the intervention period was short-term or long-term. Specifically, the short-term intervention subgroup ( < 8 weeks)—which included 11 studies—yielded a pooled standardized mean difference (SMD) of –1.14 [95% confidence interval (CI): –1.54 to –0.73; *P* < 0.00001], confirming the efficacy of brief MT. This aligns with a study on musical memory indicating that short-term MT can enhance emotion regulation and reduce anxiety symptoms by promoting dopamine secretion (Ferreri et al., 2021). Regarding MT duration, we observed that studies with ≥ 8 weeks of music therapy yielded a larger pooled effect size (SMD = –2.26) than those with < 8 weeks (SMD = –1.14). Nevertheless, the absence of post-intervention follow-up data prevented us from determining whether this difference indicated a sustained therapeutic advantage. Consequently, we could not conclude that longer treatment duration inevitably produced more durable benefits.

We also identified significant heterogeneity in the meta-analysis. Therefore, when interpreting our results, it is crucial to take into account the differences between the randomized controlled experiments. Firstly, the scales used in each study were significantly different. For example, the State-Trait Anxiety Inventory (STAI) was the most commonly used tool and was adopted by multiple studies (Newham et al., 2012; Knowles and Olatunji, 2020). Thus, the results would also vary. Secondly, there were significant differences in the participant characteristics, sample size, treatment type, and intervention duration of different groups. Finally, due to the differences in research designs, some studies included physiological indicators for assessment, such as blood pressure, pulse, blood oxygen saturation (SpO2), and vital signs, as well as cortisol levels detected through saliva enzyme-linked immunosorbent assay (ELISA). These would also lead to differences.

The extremely high heterogeneity detected in the analysis (*I*^2^ = 95 %) is unlikely to be due to random sampling error (Higgins et al., 2003). Instead, it likely reflects clinically meaningful differences between trials. First, the term “music therapy” encompasses a range of interventions, including passive, active, and combined approaches. These differ in dose, participant engagement (Bradt et al., 2010), and presumed mechanism (e.g., emotional processing, distraction, or social bonding) (de Witte et al., 2022). Therefore, their true effect sizes are not expected to be identical. Secondly, the range of baseline anxiety severity among participants also influences the results. Because music therapy may produce larger benefits when baseline distress is higher (Pelletier, 2004), this participant heterogeneity inevitably widens the confidence interval of any pooled estimate. In total, these intervention-level and participant-level “real differences” explain why the overall effect should be seen as an average across different conditions rather than a single universal estimate.

### 4.2 Strengths and limitations

This study had several significant strengths. Firstly, this study conducted a systematic review and meta-analysis. By including a total of 1,679 subjects in 18 randomized controlled trials, the scientific nature and reliability of the research conclusions were ensured. Secondly, this study adopted a combination of multi-theory model (MTM) analysis methods and randomized controlled trials (RCT), enriching the measures for research on the intervention of anxiety among college students. In addition, it verified the synergistic mechanism of the combined MT, providing new empirical support for the theoretical model of MT. Third, the results of this study have strong robustness, not only through strict literature screening and evaluation to determine the quality of the included studies, but also through effective control of confounding variables to ensure the internal validity of this study. Which were consistent with the rigorous design of important MT studies in the existing literature (Jia et al., 2025; Soqia et al., 2025).

Despite these strengths, the study has some limitations. Firstly, a high degree of heterogeneity was observed in this study, which was similar to the results of Chang et al. (2015) and van der Steen et al. (2018). This may have been attributed to differences in the MT protocol and the baseline anxiety levels of the participants. Future research will need to develop standardized research protocols to reduce significant heterogeneity results. Secondly, most studies lacked post-intervention follow-up, so this review could not determine whether the observed effects were sustained beyond the treatment period. Future studies should incorporate longer follow-up durations to assess the durability of music therapy benefits. Thirdly, active MT was underrepresented, highlighting the need for further investigation in this area. Finally, reliance on self-reported scales (e.g., anxiety questionnaires) may have introduced response bias, though this was relieved by the use of validated tools consistent with prior studies (Fan and Wang, 2001; Song et al., 2012). However, we needed to acknowledge that there was no completely satisfactory method for any meta-analysis to detect the existence of publication bias (Carter et al., 2019). Therefore, we cannot entirely rule out the existence of publication bias. Although publication bias attributed to a single large-sample study was detected in this meta-analysis, sensitivity analysis confirmed the robustness of the core findings. This indicates that the impact of publication bias on the overall conclusions is limited. Furthermore, the robustness of the results suggests that even if additional similar studies are incorporated in the future, the likelihood of reversing the core conclusions of this meta-analysis is low. These findings collectively enhance the credibility of our primary inference that Music therapy is an effective intervention for alleviating anxiety among college students.

## 5 Implications

Firstly, standardizing MT protocols: Given the high heterogeneity in existing studies, it was necessary to unify core elements such as MT duration, frequency, and music type (e.g., distinguishing between active, passive, and combined MT) to enhance the replicability and comparability of results. This aligned with the call in related research for clearer operational definitions to avoid confusion between “MT” (which requires professional guidance and therapeutic relationships) and “music medicine” (which primarily involves passive listening). Secondly, strengthening long-term follow-up studies: most current studies lacked data on sustained effects after MT cessation. Future research should extend follow-up periods to explore whether regular maintenance MT are needed to prevent anxiety recurrence, addressing the gap noted in meta-analyses regarding the durability of effects. Thirdly, expanding cross-cultural and comparative studies: while domestic studies showed significant effects, international research results were less consistent, possibly due to cultural differences in stress sources and emotional expression. More cross-cultural trials are needed to verify the adaptability of MT in diverse contexts. Additionally, comparing its efficacy with other interventions (e.g., cognitive-behavioral therapy, exercise) can clarify its unique value in mental health services. Finally, incorporating multi-dimensional assessment indicators: in addition to self-reported anxiety scales, physiological indicators (e.g., cortisol levels, heart rate variability) and neuropsychological measures should be included to provide objective evidence for the mechanisms underlying MT’s effects, enhancing the depth of research.

## 6 Conclusion

This meta-analysis confirmed the significant efficacy of MT in alleviating anxiety among college students, especially when implemented in combined formats and over longer durations. It offers a safe, low-cost, and acceptable alternative to traditional interventions such as pharmacotherapy, particularly in contexts characterized by academic pressure and “involution anxiety.” However, the use of MT in China is currently relatively limited. Future research should focus on increasing the diversity of MT methods, strengthening cross-cultural validation, and comparing its efficacy with other interventions to better integrate MT into campus mental health services. Overall, MT holds promise as a practical intervention for improving college students’ mental health, but further high-quality studies are required to refine its implementation and verify its sustained effects.
